# Accept or refuse? Factors influencing the decision-making of transplant surgeons who are offered a pancreas: results of a qualitative study

**DOI:** 10.1186/1471-2482-13-47

**Published:** 2013-10-23

**Authors:** Julika Loss, Karl P Drewitz, Hans J Schlitt, Martin Loss

**Affiliations:** 1 Medical Sociology, University of Regensburg, Franz-Josef-Strauss-Allee 11, Regensburg D-93053, Germany; 2 Department of Surgery, University Hospital Regensburg, Regensburg, Germany

**Keywords:** Pancreas transplantation, Under-utilization, Allocation process, Refusal

## Abstract

**Background:**

Most offered pancreases are not transplanted. This study investigates the factors that inform and influence the transplant surgeon’s decision to select an offered pancreas.

**Methods:**

Semi-standardized interviews were conducted with 14 highly qualified transplant surgeons from all 14 German transplant centers performing > 5 pancreas transplantations per year. The interviews focused on medical and non-medical criteria on which the individual accept/refuse decision depends. Interviews were recorded, transcribed and underwent content analysis.

**Results:**

The interviewees agreed upon certain main selection criteria, e.g. donor age, lab results, ICU stay. However, there was no consistency in judging these parameters, and clear cut-offs did not exist. The pancreas macroscopy is a pivotal factor, as well as knowing (and trusting) the donor surgeon. 3/14 surgeons reported that they had occasionally refused a pancreas because of staff shortage. Some interviewees followed a restrictive acceptance policy, whereas others preferred to accept almost any pancreas and inspect it personally before deciding.

**Conclusion:**

The assessment of medical donor characteristics is highly inconsistent. Both very cautious as well as very permissive acceptance policies may render the allocation process less efficient. A more standardized policy should be discussed. Finally, better training for donor surgeons seems advisable, in order to increase trust and thus pancreas utilization.

## Background

Whole organ pancreas-kidney transplantation has become the standard treatment for type 1 diabetes mellitus patients with renal failure [1-6]. However, not all patients eligible for pancreas transplantation can benefit from this therapeutic option, mainly because of the well-known shortage of donor organs. The waiting time for receiving a pancreas transplant has considerably increased in several European countries over the last few years [7]. In addition, the majority of pancreases that are offered for allocation are not transplanted [7]. Data from Eurotransplant (ET), an international foundation responsible for the allocation of donor organs in Austria, Belgium, Croatia, Germany, Luxembourg, the Netherlands and Slovenia, indicate that 71% of all offered pancreases are withdrawn or discarded. The reasons for this pancreas under-utilization are not yet well understood. Data from the UK indicate that the majority of organs are withdrawn from the allocation process because they are repeatedly turned down from the contacted transplant centers due to medical characteristics of the donor [8]. In Germany, data from 2005–2009 show that 43% of pancreases are withdrawn before recovery because of repeated refusals, and 20% are discarded at the time of intended recovery, or after recovery, mainly due to poor organ macroscopy [9].

When an organ is consented to be allocated for transplantation, the physician or surgeon from the transplant center with the patient ranked highest on the waiting list is contacted by phone [10]. In parallel, a recovery team in the region of the donor hospital is instructed to perform the donor surgery. The final decision whether to use the organ is prerogative of the transplant surgeon and/or physician responsible for the care of the recipient [10]. Many of these offers are turned down on the phone; data from German pancreas donors show that a pancreas is offered to a median of three centers before it is placed [9]; those pancreases withdrawn from the allocation process had been offered to a median of eight patients in five centers. This begs the questions as to which factors may influence a transplant surgeon’s decision to accept or refuse a pancreas offer. For example, it is not known whether medical characteristics of the donor are judged in a similar way by different transplant surgeons, and if so, what the underlying reasons may be. Based on the data from the allocation process in Germany, there is no consistency in the decision-making process using single donor characteristics (e.g. BMI, ICU stay, age) [9]. It has also been speculated that administrative (e.g. staff shortage) or strategic aspects (e.g. survival rates) may play a role when refusing organ offers [10]. Whether these considerations actually affect the decision-making process has not been analyzed yet. Neither has it been studied whether the decision is rather intuitive, or based upon sound evidence or consensus as to the medical criteria of the donor.

The objective of this study was to analyze

(1) Which medical donor characteristics have an impact on a transplant surgeon’s decision to accept or refuse an organ offer;

(2) Whether there are differences in evaluating medical donor characteristics between transplant surgeons of different centers with regard to acceptance of an offered pancreas;

(3) To what extent non-medical reasons, e.g. staff shortage, may play a role in the accept/refuse decision in pancreas transplantation.

## Methods

### Sample

In Germany, 163 pancreases from donors after brain death were transplanted in the year 2010 [11] (transplantation from donors after circulatory death is not allowed in Germany). These pancreas transplants were performed in 24 centers; the three hospitals with the highest volume transplanted 13, 14, and 16 pancreases per year, respectively. 14 of the centers transplanted more than five pancreases per year [11]. In all of these 14 relative ‘high volume’ centers, experienced transplant surgeons are on call to decide upon the acceptance or refusal of whole organ donor pancreases when offered by Eurotransplant. In most centers, 2–3 surgeons are specialized in pancreas transplantation.

Semi-structured, face-to-face interviews were conducted with surgical consultants from all 14 ‘high volume’ pancreas transplant centers between May and November 2011). The interview partners (n = 14) met the following inclusion criteria:

employment at a transplant center performing >5 pancreas transplantations per year (according to 2010 data)

minimum of three years of experience in pancreatic transplant surgery

routine responsibility for decisions on accepting or refusing offered donor pancreases

Purposive sampling was used to recruit the sample. Two transplant surgeons from transplant centers with less than five pancreas transplants per year were interviewed first in order to pre-test the interview guide; they provided comments on the questions (for the final interview guide see Table 1). These interviews were not included in the analysis. All participants agreed to being interviewed and recorded. The transcript, analysis and presentation of data did not enable identification of individual participants or their respective centers.

**Table 1:** Interview guide

**Introductory questions**	• How long have you been working as a transplant surgeon in a responsible position?
	• Are you currently performing pancreas recoveries as well?
	• If a pancreas is offered to your center, what is the usual process?
**Broad topic 1: Deciding about a pancreas offer**	• When deciding about a pancreas offer from Eurotransplant, what criteria guide your decision?
	- *If medical/social parameters that are interval variables (e.g. age, BMI, length of ICU stay, P-PASS*^1^*) are mentioned:* Do you apply certain cut-offs for this parameter?
	• How relevant is the macroscopic evaluation performed by the recovery team?
	• How important is it for you that the donor and recipient match well?
**Broad topic 2: Non-medical refusal reasons**	• How might your decision be influenced if you are told that the organ has been previously refused by other centers?
	• Have you ever heard that pancreases have been refused due to capacity reasons or even witnessed it personally?
	• Could you imagine that under certain circumstances, a pancreas might be refused due to infrastructure reasons in your clinic, e.g. due to staff shortage? Has that ever happened?

^1^P-PASS = pre-procurement pancreas allocation suitability score, combining nine donor parameters, e.g. age, body mass index, ICU stay, and (nor)adrenaline use. The DRI (donor risk index), an alternative prognostic score for pancreas transplantation, is not commonly used in Germany and was therefore not included explicitly in the interview guide.

### Data collection

The interviews covered factors that influence the transplant surgeon’s decision to accept or refuse a pancreas. If donor-related medical factors such as age, BMI or length of ICU stay were named by the interview partner, it was asked whether certain limits would apply for these variables, and what these limits were. If non-medical criteria were not mentioned spontaneously, e.g. staff shortage, these aspects were asked explicitly. One of the authors (KPD) conducted all the interviews, each of which was audio-taped and lasted for 20–70 minutes.

### Analysis

Interview tapes were transcribed verbatim. The transcripts were de-identified and continuously numbered (Interview Partner = IP 01-IP 14), so none of the researchers, except the interviewer, could link the answers back to the interviewed transplant surgeon or the respective transplant center. The transcripts were examined using thematic content analysis [12]. Key themes that spanned specific questions and topics were identified, and were used to organize the material [13]. In the course of the data analysis, the initial categories, distinguishing between medical and non-medical factors influencing the decision, evolved into more sophisticated coding structures and additional categories, including e.g. concepts of trust, patient’s prognosis, and strategic aspects. To enhance the validity of the findings, the interview transcripts were read and coded independently by two authors (KPD and JL); deviant cases and contradictory data were analyzed with particular attention [14].

### Ethical approval

No authorized ethical approval was needed as per the ethics committee of the University of Regensburg (reference number 11-160-0192).

## Results

The 14 transplant surgeons reported to be performing transplant surgery in authoritative positions for 3–15 years. All interview partners with the exception of one reported to be routinely involved in donor surgery of the pancreas as well, being a member of a regional recovery team. According to the interview partners, transplant surgeons have the sole responsibility of the accept/refuse decision for offered pancreases in 11 transplant centers, whereas a joint decision between transplant surgeons and nephrologists is the usual procedure in three transplant centers.

**Figure 1:**
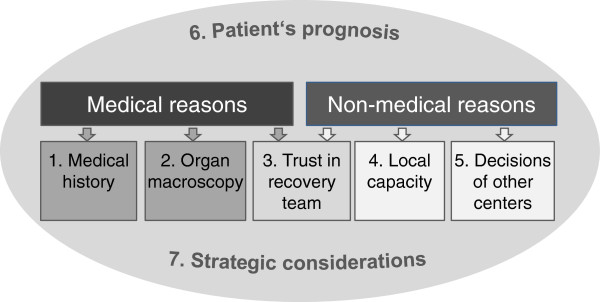
Categories of criteria that play a role when deciding to accept/reject a pancreas.

As illustrated schematically in Figure 1, we identified seven main factors influencing the decision-making process (the numbers in brackets refer to Figure 1 and to the subheadings of the following text): The decision to accept or refuse an offered donor pancreas is mainly based on medical criteria. The key factors of medical criteria are the donor’s medical history (1), and, significantly more important, the donor organ macroscopy (2). The decision-making process is also influenced by confidence in the recovery team’s expertise (3). Non-medical aspects play a role in certain circumstances, mainly in terms of staff shortage (4) or prior decisions of other centers (5). The decision is also influenced by the comparatively benign prognosis of patients waiting for a pancreas transplant (6). Finally, the decision-making process can also be guided by strategic considerations, e.g. regarding competition with other centers, or risk management (7).

1. Donor medical history

All interview partners mentioned at least 3–6 donor characteristics that are considered decisive for evaluating a pancreas offer. Some of these characteristics were named by almost every interview partner: age, length of ICU stay, cause of death, lab results, and co-morbidities. Other characteristics, such as BMI, history of alcohol abuse, or administration of blood transfusions were named less frequently. When asked about resuscitation of the donor, the majority of interview partners denied this criterion to be of importance.

While there was a broad consensus on the donor characteristics that are relevant to evaluate a pancreas offer, the cut-offs used for some of these characteristics varied substantially between interview partners.

*Donor age.* Donor age plays a prominent role for the evaluation of the pancreas. The upper age limit which was considered acceptable ranged from 40 to 55 years of age. The majority of interviewed surgeons pointed out that the donor age needed to be judged in relation to other donor characteristics: the more risk factors were present, the less tolerable is an age above 45 or 50 years.

With a 50-year-old, you need to check what other factors there might be…How is his/her glucose level? Does co-morbidity play a role? Does the offered organ appear to be from a biologically younger donor? (IP11).

Two surgeons, however, reported to have fix cut-offs for donor age.

We have relatively strict guidelines… It starts already with age. As a rule, we do not accept organs that are more than 40 years old. (IP 06)

*Length of ICU stay*. Most interview partners explained that they appraised the length of ICU stay in relation to other donor characteristics; however, there was a wide range of rough cut-offs mentioned (from 5 days to 2–3 weeks up to 20–30 days).

[The length of ICU stay] should not exceed a week. If it’s two and half to three weeks, I would be skeptical about the organ. (IP 10)

[I don’t draw] a line there. There are patients who have stayed in the ICU for three or four weeks. With this deadline of one week and then rolling their eyes, saying: ‘Oh, 10 days already!’ For us, this is nonsense; it’s no reason to turn down an organ. (IP 05)

*Lab results.* The majority of interviewed surgeons reported to routinely check the laboratory values of the donor, but none named concrete limits. The donor’s lab results are only one of many aspects that influence the accept/refuse decision. Some surgeons stated that lab results play a minor role compared to the organ macroscopy.

*Interdependence of donor characteristics.* Most surgeons explained that as a rule, none of the mentioned donor criteria alone justified an organ refusal; different factors need to be weighed against each other. Organs may be declined for a number of relative risk factors. It became clear, throughout the interviews, that different factors were not assessed in relation to each other in a systematic process, but very individually and intuitively for every donor.

The decision is cumulative: old age, pancreatitis, poor circulation, and then - on top of that - maybe overweight. Then you add it all up. But refusing it [the organ] just because of a single criterion, we wouldn’t do that. (IP 05)

The refusal reasons that are given to the ET, these are just an accumulation of bad gut feelings. (IP08)

*P-PASS*: The P-PASS, a score system combining several risk factors of the donor, does not serve as a decision aid; four out of 14 interview partners mentioned that they use it, but explained that its significance was either limited or decreasing in importance.

*Matching between donor and potential recipient.* The majority of interview partners stated that it is not crucial that the donated organ matches well with the potential recipient, e.g. in terms of size – other than e.g. in liver transplantation.

*Evidence base*. Although not explicitly asked in the interview, the surgeons expressed that their criteria and cut-offs are based on various grounds, as presented in Table 2.

2. Organ macroscopy

The surgeons stated unanimously that the macroscopy of the pancreas is the key parameter for judging its quality. Many surgeons described it as superior to information about the donor’s general medical condition for the assessment of the pancreas quality.

If the CRP is a little high, it doesn’t mean that you can’t transplant [the pancreas]. It’s the same with lipase: as long as it isn’t above 1000 [U/l], it doesn’t bother me. If it [the pancreas] is macroscopically fine and recovered well, you would certainly transplant it. (IP 09)

For me, the surgical parameters are more important, but the nephrologists primarily look at age, lab results, sodium, amylase, … creatinine, glucose (…). I rather look at the parenchyma (…), vascularization [and the] cold ischemic time. (IP 07)

3. Confidence in recovery team

*Pancreas macroscopy as viewed by recovery team.* Normally, the transplant surgeons who need to decide to accept an organ have to rely on the donor surgeon’s judgment of the organ macroscopy, often communicated via phone. If the recovery team describes the pancreas quality as poor, transplant surgeons react differently, according to the interviews, as shown in Table 3. Trust plays an important role in this context.

The policy of eagerly accepting offered pancreases with the aim to inspect the organs personally (Table 2, 2nd category) is explicitly criticized by one interviewee. This approach might decrease the organ’s probability to be transplanted elsewhere in case of refusal, due to overlong cold ischemic time.

There are certainly centers that accept such an organ just so that they can have it and, more or less, take it off the market for any other center. And then in the end, [they] decide not to transplant it. And eventually, with this approach, you won’t be able to transplant it at all. You experience that every day basically. This is definitely the wrong trend and you have to work against it. And if you …start that too,…in order not to fall behind, then this spiral will keep on going. It’s the same as in the 1980s with the arms race between the USA und Soviet Union …“. (IP02)

*Technical quality of pancreas recovery.* Almost all interviewed consultants agreed that the success of the transplantation is dependent upon a skilled organ recovery. In principal, this factor cannot influence the accept/refuse decision prior to actually receiving the accepted organ in the transplant center; however, there were hints that a lack of confidence in the donor surgeon’s competence might influence the decision to accept an organ.

For me, it’s essential to have a good donor and an excellent recovery. That‘s why I‘m convinced […] that you can say that your reason for refusal was that the recovery surgeon had no clue. And I‘m one of the few people who gives this as the reason. (IP04)

4. Capacity of transplant center

The vast majority of the interviewed transplant surgeons was convinced that transplant centers in Germany occasionally turn down pancreas offers due to staff shortage. However, nine out of 14 clearly ruled out that option for their own respective hospitals, declaring that refusals on the grounds of staff shortage were ‘nonsense’ or ‘a non-issue’. According to the interview partners, the main reason for capacity problems is a limited number of experienced transplant surgeons in a hospital, coinciding with sickness, holidays or scientific conferences. Three interviewed surgeons admitted that they had already refused organs for capacity reasons.

I’m with a center now where this [capacity] problem is not really the issue. Because we’ve spread ourselves out pretty well…So there’s always someone there who is available and who is able to transplant a pancreas. I could certainly imagine though, that it’s different in smaller centers, because I already know how difficult it is for us to organize ourselves this way. (IP 11)

I have already refused organs because it simply wasn’t possible, logistically. On Easter Sunday, an organ offer arrives, and the surgeon on call is sick. Then I have to refuse the organ for organizational reasons. (IP 07)

We inform all potential recipients on the waiting list about the possibility of a capacity shortage – for whatever reasons: ICU bed, surgeon, other things… I have already cancelled organs due to capacity problems. We communicate this to our patients in a transparent way. (IP 08)

Whereas two interview partners emphasized that it was the obligation of a transplant center to have the necessary resources for transplantation available at any time and under any circumstances, others (6/14) described capacity problems as unavoidable or even legitimate.

If you provide a transplant program in your hospital, you have to make sure that the organs that come in can be transplanted. […] You also have the responsibility for the patient, who has been on the waiting list for years. And then say, ‘Oh, there were three others in parallel and there was only one surgeon who had to take care of all of them” – the patient on the waiting list will not understand that.’ (IP05)

[A transplant surgeon] is allowed to get sick, to go on holiday…, meaning that he can‘t [operate]. And I can very well imagine that someone says, due to logistical reasons [they can‘t do it]. And this shouldn‘t be seen as a reason to stigmatize these people. (IP04)

It transpired in the interviews that transplant surgeons might be led into using medical reasons as a pretext when an organ needs to be refused for organizational reasons.

5. Previous turn-downs by other centers

If the pancreas had previously been offered to other transplant centers by Eurotransplant and had been refused, it was of little to no relevance for most interview partners.

We always look at the organ individually. You can’t imagine what we have experienced – pancreases are refused for the most diverse reasons. We always want to see for ourselves what’s there. (IP 12)

In four out of 14 interviewed surgeons, however, there was a tendency to consider or to at least be influenced by the decisions of other transplant centers.

I ask what was the reason [for turning down the organ]. This is for me, above all, a time-saving factor. When I get a phone call at 3 a.m., and they tell me there had already been 3 centers. And these 3 centers are certainly not that stupid, they must have had some reason. (IP 08)

As a rule, a good organ is accepted by the first center. If it isn’t accepted, this is already sort of suspicious. (IP 07)

6. Patient’s prognosis

The interviewed surgeons pointed out that a pancreas transplant differs from a liver, heart or lung transplant in that the indication is, as a rule, not vital. Therefore, the organ needs to be selected more carefully, because the willingness to compromise is smaller than in cases of life-threatening conditions. This may lead to a cautious acceptance policy.

If I am in doubt, I’d rather refuse. Simply because the recipients are so selected. You can hardly expect them to put up with the possibility that it doesn’t work well afterwards […] If you don’t transplant the recipient, the chance that he or she dies is small. If you give him or her a bad organ, the chance that he or she dies is actually given. You simply have to consider that. (IP 08).

The waiting time …for pancreas-kidney-transplants in Germany is… not so extreme that you need to rush someone into transplant surgery…I cannot endanger a human being in order to perform a transplant when I know that an organ from a good pool will arrive within the next 18 months anyway. (IP 13)

This flexibility in accepting or turning down an offered pancreas is accentuated by the lack of clear standards and cut-offs; therefore almost any pancreas can be refused.

And most of the donors do have some flaw…. And then this one flaw can be made into a big deal, and that’s why we only utilize only [so few] of the offered organs. And then there is the question: how many flaws do you want to accept? If you don’t want to run any risks, then you don’t accept any flaw. (IP 03)

7 Strategic aspects

Some further factors which relate to expectations of the hospital administration or aspects of competition (with other transplant centers) might also influence the decision making process, although this was only hinted at in few interviews.

Here, we need to justify ourselves very well, in the clinic and to our boss, and need to have good reasons if we turn down such an offer. (IP 05)

In addition, younger surgeons who are inexperienced or surgeons from a center with a higher rate of complications might decline an organ offer in order not to risk a surgical failure, as two interviewees surmised.

A lot of this is about experience. A center that, for example, has had many complications will certainly be more restrictive. And when there’s a tiny little thing [about the pancreas], they say, for safety reasons, that they refuse it. A center with more experience and fewer complications will surely loosen its criteria…Experience plays a major role there. (IP 09)

**Table 2:** Basis of the assessment of medical donor characteristics

**On which basis do transplant surgeons assess medical donor characteristics?**		
***Category***	Frequency*	***Sample quote***
1) International literature	3/14	*With the pancreas, I actually stick to the published data from Minneapolis, that is to Sutherland [et al.], on risk factors for organ loss…to this study with more than 1,000 pancreas transplantations. (IP 02)*
2) Hospital guidelines	2/14	*We have relatively strict guidelines…It starts already with age. As a rule, we do not accept organs that are more than 40 years old. Institutional guidelines. (IP 06)*
3) Peer custom	2/14	*There’s this age limit‚ 50 years‘. Wherever that may come from. (IP 12)*
		*[I check] donor age, cause of death,…weight. These are the essential [factors]. I guess this is generally valid, everywhere. We don’t have special [age] regulations [in our center]. (IP 08)*
4) Personal clinical experience	4/14	*When I look back on our most recent pancreases, they were all between 45 and 55 years [old]. …I think we can do it, transplanting these organs, because we don’t have these long shipping times [like in the USA],… we are very successful at it.(IP 05)*
		*Polytrauma… whoever refuses that per se, but accepts a 40- or 50-year-old, who has seen younger days – my personal opinion is that this is wrong. (IP 11)*

*The numbers do not add up to 14, because not every interview supplied sufficient information to allow for a clear categorization. In addition, 2 interviewees were coded for 2 categories, because they relied on the scientific literature, but also considered their clinical experience.

**Table 3:** Handling the pancreas assessment of the recovery team

**If the recovery team describes the pancreas quality as being poor, transplant surgeons react differently:**			
***Category***		Frequency*	***Sample quote***
1)	The interviewee adopts the assessment of the recovery team	3/14	*If [the donor surgeon] says: ‘It’s obviously hardened’, then you needn’t discuss it, then it is hardened. (IP 09)*
2)	The interviewee adopts the assessment only if he knows and trusts the recovery surgeon	3/14	*There’s a matter of trust involved here. […] We know that people who have never seen (or) performed pancreas transplantations in their lives are recovering these organs. I think that’s difficult… the foundation of trust is missing there. If I know that this and that person has recovered the organ, [and that] he knows what he is doing, then I can …say l’ll take it. (IP 14) When I am informed that [the donor surgeon] is working with a center that has never had a pancreas transplant program, then, honestly speaking, I’m very sceptical… A macroscopic assessment from somebody who isn’t even familiar with it [pancreas transplant], you can completely forget that. (IP 08)*
3)	The interviewee does not rely on the assessment of the recovery team. He prefers to accept the organ in (almost) any case and assess it personally	6/14	*[Assessment of the donor surgeon] - I know, from experience, that there are people who have no idea whatsoever what a pancreas should look like. So as a rule I consider [their assessment] for my decision, [but] unless there’s an injury or something that clearly precludes [a transplantation], … I’d rather look at it personally. (IP 12) [We accept] even if the recovery team informs us that the organ is fatty or hard – if the parameters on paper are okay. (IP 05)*

*The numbers do not add up to 14, because not every interview supplied sufficient information to allow for clear categorization.

## Discussion

### Principal findings

From the interview results we could gain insight into the decision-making process of transplant surgeons who are offered a pancreas: The main factors that influence the decision (e.g. pancreas macroscopy, donor medical history) are relatively consistent throughout the interviews. However, when analyzed in depth, there are extreme differences between the surgeons’ assessment and general handling of pancreas offers. Firstly, while nearly all of interviewed surgeons list the same medical donor factors as being relevant for pancreas selection (above all donor age, time of ICU stay, and lab results), the assessment of these factors varies substantially between the surgeons; no standardized or consistent cut offs exist. For example, some surgeons told the interviewer that they had no doubts about accepting the pancreas of a 55-year old if the other parameters were favorable, whereas others claimed not to accept the pancreas of a donor over 40.

Secondly, the macroscopic appearance of the donor organ and the technical quality of the pancreas recovery are the predominant factors which influence the decision. In Germany, the transplanting surgeons usually do not perform the pancreas recovery as well, so the macroscopy is described to them on the telephone. The transplant surgeons differed significantly as to whether or not they relied on the donor surgeon’s assessment of the macroscopic pancreas quality. Some prefer to have the organ shipped to their center even if it has been described as having poor quality. Others trust the donor surgeon’s judgment – some surgeons do so in every case and others only if they know and have confidence in the respective donor surgeon.

Non-medical factors play a minor role, but certainly exist; above all, capacity problems can lead to the refusal of pancreases in some centers. Interestingly, the sample was split on the legitimacy of occasional staff shortages.

It became clear in the interviews that the relatively benign prognosis of the patients on the pancreas waiting list may induce surgeons to wait for excellent (‘flawless’) organs. This restrictive policy is in contrast to some surgeons’ preference to accept pancreases in a very permissive way – even if turned down frequently before and having been described as macroscopically poor – so that they can inspect and evaluate the organ personally.

### Strengths and weaknesses of study

Although the phenomenon of pancreas under-utilization is well known in European and North American transplantation networks and has been described as a ‘major issue in pancreas transplantation’ [15], the analysis of the underlying reasons has been only superficial thus far. The strength of this study lies in the investigation of one of the pivotal steps in the allocation process: the transplant surgeon’s decision to use or not to use an offered pancreas. The main limitation of the study is that the interview partners were recruited by purposive sampling, and were limited to a number of 14 interview partners. Consequently, it cannot be assumed that the findings presented here are representative of the views of all pancreas transplant surgeons in Germany. However, the aim of qualitative studies is not to receive representative data, but to gain a deeper understanding of social and psychological processes. Using a qualitative study design allowed us to tap personal attitudes and experiences that are not readily expressed in response to survey questions [16], and that are key to understand the complex processes and conditions involved in making the decision. While the number of interviewed transplantation surgeons was relatively small, the selected interview partners represented all German transplantation centers that perform five or more pancreas transplantations per year, thus constituting a very balanced and almost representative sample. The use of the same interviewer for all participants, and two independent researchers for data analysis ensured quality control and minimal interpretive bias.

### Comparison with other studies

In order to explain the phenomenon of pancreas under-utilization, few quantitative studies analyzed donor characteristics of transplanted and/or discarded pancreases. Wullstein et al. compared donor profiles of accepted pancreas grafts versus grafts declined due to ‘medical reasons’. They found significant differences between both groups for cause of death, age, BMI, serum Lipase, alcohol abuse and history of smoking. They concluded that these aspects might be the most important reasons to refuse a pancreas [17]. These medical characteristics are largely overlapping with those named by the interviewed surgeons, although Wullstein et al. had not included the length of ICU stay in their analysis, which was a predominant aspect in the interviews. However, our analysis shows that the reasons to accept or refuse an offered pancreas cannot be pinpointed to single donor characteristics, but are far more complex. Wiseman et al. [18] analyzed multiple characteristics of donors whose pancreas was used, and compared them to donors whose pancreas was not used. This study found that a significant number of potentially suitable donor pancreases were not used, although no medical characteristics that precluded transplantation were identified. In the article’s discussion, Wiseman et al. speculated that ‘transplant centers … may be reluctant to accept pancreases that are not assessed by members of their own team or by colleagues who have demonstrated experience in pancreas transplantation.’ This notion by Wiseman, which was not backed by any other data yet, could clearly be confirmed by our study. Wiseman et al. also hypothesized that due to the relatively short waiting time, ‘the tendency for pancreas programs to decline use of a pancreas …with the hopes that an even more “optimal” donor could be forthcoming in the near future may limit pancreas utilization’. The finding of the interviews that some hospitals or surgeons display a stringent acceptance policy is consistent with this supposition.

### Meaning of the study/policy implications

The interviews shed light on some aspects that help explain the under-utilization of donor pancreases.

It became clear that transplant surgeons who decide to accept or refuse a pancreas offer always act on conflicting priorities: on one hand, the expectations of patients and hospital administration not to turn down a rare and precious donor organ; and on the other hand, the fear of endangering the patient’s health by accepting an organ that is not flawless. The latter is especially important in pancreas transplantation because unlike liver or heart transplantation, the patient’s condition is usually not life threatening, so there is less willingness to compromise. If many centers dictate a cautious acceptance policy, organs might be refused repeatedly. Consequently a ‘cascade effect’ [19] can ensue, because the refusal of one center might - consciously or unconsciously - increase the probability of further refusals, as the interview analysis suggests. This is especially critical if the allocation process is still ongoing when the organ has already been recovered. As a consequence, the extended ischaemic time may result in an increase of discarded organs; it can also lead to unequal access to donated pancreases [20]. Conversely, a very permissive acceptance policy that some interview partners displayed might also lower the pancreas utilization rate, because there is the risk involved that the organ cannot be placed anymore if its macroscopy is not considered favorable when the transplant surgeon inspects the organ personally in his center.

The interview results suggested that the assessment of medical donor characteristics is subjective, inconsistent and hardly standardized. This observation can also be explained by the fact that the interviewed surgeons base their cut-offs on varying evidence or customs. A more standardized approach in terms of cut-offs seems to be difficult, however, because one parameter needs to be assessed in conjunction with other risk factors. One has to discuss whether the allocation process would profit from a better standardization or an evidence-based approach. Particularly younger surgeons who have less experience might benefit from recommendations or guidelines, which can be developed by experts. However, one needs to consider that there is still incomplete evidence on whether or not a pancreas is suitable for transplantation, as recently pointed out by Magione et al. [21].

Within the relatively small community of surgeons who are experienced in pancreas transplantation, donor surgery and pancreas surgery, the aspect of trust seems to be an influential factor in the decision-making process: confidence in the donor surgeon and his or her capacity to a) assess the pancreas quality and b) to recover a pancreas without damaging it, and maybe even trust in the transplant center that had turned down the organ offer prior to one’s own decision. Better and more standardized training or perhaps more rigorous selection of donor surgeons could improve the expertise and thus boost confidence.

## Conclusion

The assessment of medical donor characteristics is highly subjective and inconsistent. Both very cautious as well as very permissive acceptance policies may render the allocation process less efficient. A more standardized policy should be discussed. Finally, better training for donor surgeons seems advisable, in order to increase trust and thus pancreas utilization.

## Endnote

^a^P-PASS = pre-procurement pancreas allocation suitability score, combining nine donor parameters, e.g. age, body mass index, ICU stay, and (nor)adrenaline use. The DRI (donor risk index), an alternative prognostic score for pancreas transplantation, is not commonly used in Germany and was therefore not included explicitly in the interview guide.

## Competing interests

The following authors of this manuscript have conflicts of interest to disclose: ML has received consulting fees and lecture fees from Novartis, Astellas, Genzyme, Roche, and Biotest. HJS has received honorarium for lectures, advisory boards, data monitoring committees (DMCs) and research support from Novartis and Roche, honorarium for advisory boards from Bristol-Myers Squibb, and research support from Pfizer. JL has received support to her institution from Novartis in order to cover the travel expenses for KPD.

## Authors’ contributions

ML and JL had the idea for the study, developed the research design and methods, participated in data analysis and wrote the manuscript. KPD performed the data collection and analyzed the data. HJS contributed to developing the research design, was involved in the discussion of the analysis, and contributed to writing the manuscript. All authors read and approved the final manuscript.

## Pre-publication history

The pre-publication history for this paper can be accessed here:

http://www.biomedcentral.com/1471-2482/13/47/prepub
